# Comparative Study of Endoscopic Transcanal Tympanoplasty and Tympanoplasty by Conventional Postaural Approach in a Tertiary Care Hospital in Central India

**DOI:** 10.7759/cureus.67081

**Published:** 2024-08-17

**Authors:** Jasleen Kaur, Prasad T Deshmukh, Sagar S Gaurkar, Shraddha Jain, Ayushi Ghosh Moulic, Parindita Sarmah, Vaibhavi Patil, Abhijeet Sharma, Aashita Malik, Venkat Reddy

**Affiliations:** 1 Otolaryngology - Head and Neck Surgery, Jawaharlal Nehru Medical College, Datta Meghe Institute of Higher Education and Research, Wardha, IND; 2 Pediatrics, Jawaharlal Nehru Medical College, Datta Meghe Institute of Higher Education and Research, Wardha, IND; 3 General Medicine, Jawaharlal Nehru Medical College, Datta Meghe Institute of Higher Education and Research, Wardha, IND

**Keywords:** chronic otitis media, hearing loss, platelet-rich plasma, postaural tympanoplasty, endoscopic tympanoplasty

## Abstract

Background

Chronic otitis media (COM) often necessitates tympanoplasty to repair the tympanic membrane. While conventional postaural tympanoplasty (PA) is well-established, endoscopic transcanal tympanoplasty (ET) is gaining traction for its minimally invasive benefits. This study aims to compare these two surgical techniques regarding their anatomical and functional outcomes and assess the role of platelet-rich plasma (PRP) in improving these outcomes.

Material and methods

This prospective comparative study was conducted at Acharya Vinoba Bhave Rural Hospital, involving 60 patients with COM. Participants were randomly assigned to receive either ET or PA, with each group further subdivided based on PRP use. Preoperative evaluations included auditory function tests and diagnostic endoscopy. Postoperative assessments were performed at seven days, one month, and three months to evaluate graft acceptance and hearing improvement using pure tone audiometry (PTA). Statistical analyses included the chi-square test, t-test, ANOVA, and paired t-test.

Results

The study included patients with a mean age of 38.1 years, predominantly female (71.67%). ET demonstrated superior anatomical outcomes compared to PA, with higher graft acceptance rates and better hearing improvements. The average hearing gain was 10.4 dB in the ET group versus 8.1 dB in the PA group. PRP uses enhanced graft acceptance and hearing restoration across both surgical approaches, contributing to better overall outcomes.

Conclusion

ET offers significant advantages over conventional postaural tympanoplasty in terms of anatomical and functional results. PRP further improves surgical outcomes, making ET a preferable option for tympanoplasty in COM patients. These findings support the broader adoption of ET and PRP to enhance patient outcomes in tympanoplasty procedures.

## Introduction

Chronic otitis media (COM) is a prevalent condition characterized by persistent middle ear and tympanic membrane inflammation, often leading to hearing loss and recurrent infections [1]. Tympanoplasty, the surgical repair of the tympanic membrane, is a standard intervention for managing COM, primarily aiming to improve hearing and prevent recurrent infections [2]. Historically, tympanoplasty has been performed using the conventional postaural approach, which involves a behind-the-ear incision to access the middle ear. This method allows for a direct view of the tympanic membrane and middle ear structures but can be associated with longer recovery times and visible scarring [3]. Despite its effectiveness, the conventional approach may not always be optimal for all patients.

Endoscopic transcanal tympanoplasty (ET) has emerged as a less invasive alternative in recent years. ET utilizes endoscopic techniques to perform the procedure through the ear canal, avoiding the need for external incisions. This approach offers advantages such as reduced postoperative pain, shorter recovery times, and minimal scarring [4]. However, the effectiveness of ET compared to the conventional postaural approach remains a subject of ongoing research. Platelet-rich plasma (PRP) has been introduced as an adjunct to tympanoplasty to enhance graft healing and improve surgical outcomes. PRP, derived from a patient's blood, contains growth factors that may promote faster tissue regeneration and better graft integration [5]. The impact of PRP on the outcomes of both ET and conventional tympanoplasty is yet to be fully elucidated.

Several studies have compared the outcomes of endoscopic versus conventional tympanoplasty, but results have been inconsistent. Some studies suggest that ET provides comparable or superior anatomical and functional results compared to traditional methods [6-8]. Others have highlighted the need for further investigation into specific patient factors and surgical conditions that might influence outcomes [9]. Understanding the comparative effectiveness of these two surgical approaches, especially with the integration of PRP, is crucial for optimizing treatment strategies for patients with COM. This study aims to fill the gap in the literature by providing a detailed comparison of ET and conventional postaural tympanoplasty in a tertiary care setting, focusing on anatomical and functional outcomes.

## Materials and methods

Study setting

The study was conducted at Acharya Vinoba Bhave Rural Hospital (Datta Meghe Institute of Higher Education and Research), a tertiary care facility with the necessary infrastructure for performing advanced tympanoplasty procedures. The hospital's ear, nose, and throat department provided a suitable environment for ET and conventional postaural approach surgeries. The study adhered to all institutional protocols and ethical guidelines.

Study design

This investigation was designed as a prospective comparative study. The primary aim was to evaluate and compare the functional and anatomical outcomes of two different tympanoplasty techniques: ET and tympanoplasty via the conventional postaural approach. The study followed a structured timeline, starting with patient selection through preoperative evaluations and surgical interventions and concluding with postoperative follow-ups.

Data collection

Patient Selection and Baseline Data

The study enrolled 60 patients diagnosed with COM who met the inclusion criteria and provided informed consent. Each patient underwent a comprehensive preoperative assessment to establish baseline data. This assessment included a detailed history and physical examination focused on ear symptoms and overall health. Routine blood investigations, including a complete blood count and liver and renal function tests, were performed to rule out underlying health issues. Auditory function was evaluated using tuning fork tests at 256 Hz, 512 Hz, and 1024 Hz [10]. Pure tone audiometry (PTA) was conducted with the ALPS AD 2000 machine to determine the degree of hearing loss [11]. Additionally, diagnostic nasal endoscopy (DNE) with a 0° and 30° rigid Karl Storz nasal endoscope was employed to assess nasal and throat conditions. At the same time, examination under a microscope using the Karl Kaps D 35614 Asslar Europa Strasse microscope provided detailed views of the ear canal and tympanic membrane [12].

Surgical Intervention

Based on the surgical approach, patients were divided into two main groups: Group 1 underwent ET, and Group 2 had conventional postauricular tympanoplasty. Each group was subdivided based on whether PRP was used during surgery. The surgical procedures followed the specific techniques for each approach, with meticulous documentation of intraoperative findings. PRP preparation involved blood collection, centrifugation to separate plasma and platelets, and applying the prepared PRP during the surgical procedures.

Postoperative Follow-Up

Postoperative follow-ups were scheduled at seven days, one month, and three months. Graft acceptance was evaluated through visual inspection and endoscopic examination to ensure proper healing and integration of the graft. Hearing improvement was assessed using PTA at the three-month follow-up to measure changes in hearing thresholds. Preoperative, intraoperative, and postoperative images were captured to document the outcomes and provide visual evidence of the surgical results.

Statistical analysis

Descriptive statistics were employed to summarize the demographic and clinical characteristics of the study participants. For continuous variables, such as age and duration of illness, we calculated the mean, median, standard deviation, and range. Categorical variables, including gender and type of surgery, were analyzed using frequencies and percentages to provide a detailed overview of the patient population. Various statistical tests were applied to compare outcomes across different groups. The chi-square test was used to analyze categorical variables, while the independent t-test was employed to compare continuous variables between independent groups. For comparisons within the same group, the paired t-test was utilized. When comparing more than two groups, ANOVA was conducted, followed by post-hoc tests to pinpoint specific group differences when significant results were observed. An alpha level of less than 0.05 was established as the threshold for statistical significance, with analyses performed using SPSS software (IBM SPSS Statistics for Windows, IBM Corp., Armonk, NY) [13].

Ethical considerations

The Institutional Review Board (IRB) or Ethics Committee of Datta Meghe Institute of Higher Education and Research approved the study, ensuring compliance with ethical standards and guidelines for clinical research. Written informed consent was obtained from all participants before their inclusion in the study. The consent process provided detailed information about the study’s purpose, procedures, potential risks, and benefits, ensuring that participants made an informed decision about their involvement. Patient data were handled with the utmost confidentiality. Identifiable information was anonymized or de-identified in all reports and publications to protect patient privacy and ensure compliance with data protection regulations.

## Results

In this study, the ages of the sample range from 14 to 72 years, with a mean age of 38.1 years and a standard deviation of 14.7 years. Among the patients, 43 (71.67%) are female and 17 (28.33%) are male. Concerning perforation size, 11 (18.33%) have small perforations, 25 (41.67%) have moderate perforations, and 18 (30%) have large perforations. Table 1 shows that most patients undergoing tympanoplasty are aged 19-50 years, with endoscopic tympanoplasty being more common in the 19-35 age group and postauricular tympanoplasty in the 36-50 age group. Older patients (66+) are least represented.

**Table 1:** Age-wise distribution of patients

Age group	Endoscopic tympanoplasty	Postauricular tympanoplasty	Total
0-18	4 (13.3%)	0 (0.0%)	4 (6.6%)
19-35	11 (36.7%)	9 (30.0%)	20 (33.3%)
36-50	9 (30.0%)	14 (46.7%)	23 (38.3%)
51-65	3 (10.0%)	5 (16.7%)	8 (13.3%)
66+	3 (10.0%)	2 (6.7%)	5 (8.3%)
Total	30 (100%)	30 (100%)	60 (100%)

Table 2 shows a significant correlation exists between hearing loss and perforation size (p = 0.001). Mild hearing loss is associated with small perforations, while moderate hearing loss correlates with moderate perforations.

**Table 2:** Correlation between degree of hearing loss with size of perforation p-value: significant value

Hearing loss	Small	Moderate	Large	Subtotal	Total	p-value
Mild	8 (33.3%)	8 (33.3%)	6 (25%)	2 (8.33%)	24 (40%)	0.001
Moderate	3 (9.68%)	15 (48.3%)	11 (35.4%)	2 (6.45%)	31 (51.6%)	
Severe	0 (0%)	2 (40%)	1 (20%)	2 (40%)	5 (8.3%)	

Table 3 shows endoscopic tympanoplasty is preferred for moderate hearing loss, whereas postauricular tympanoplasty is more common for mild hearing loss. Severe hearing loss is less frequent and is mainly treated with endoscopic tympanoplasty.

**Table 3:** Distribution of patients according to amount of hearing loss and surgical approaches

Hearing loss	Surgery type	Total
Mild	Endoscopic tympanoplasty	10 (16.7%)
	Postauricular tympanoplasty	14 (23.3%)
Moderate	Endoscopic tympanoplasty	17 (28.3%)
	Postauricular tympanoplasty	14 (23.3%)
Severe	Endoscopic tympanoplasty	3 (5.0%)
	Postauricular tympanoplasty	2 (3.3%)
Total		60 (100.0%)

Table 4 shows PRP usage varies with hearing loss and surgical approach. It is less used in mild hearing loss and more in moderate cases, particularly with endoscopic tympanoplasty.

**Table 4:** Distribution of patients according to the amount of hearing loss and surgical approaches with PRP PRP: platelet-rich plasma

Hearing loss	Surgery type	PRP not used	PRP used
Mild	Endoscopic tympanoplasty	7 (70.0%)	3 (10.0%)
	Postauricular tympanoplasty	9 (64.3%)	5 (16.7%)
Moderate	Endoscopic tympanoplasty	8 (47.1%)	9 (30.0%)
	Postauricular tympanoplasty	6 (42.9%)	8 (26.7%)
Severe	Endoscopic tympanoplasty	0 (0.0%)	3 (10.0%)
	Postauricular tympanoplasty	0 (0.0%)	2 (6.7%)
Total		30 (50.0%)	30 (50.0%)

Table 5 shows PRP is more frequently used for small and moderate perforations. The usage is balanced but slightly higher for smaller perforations.

**Table 5:** Distribution of patients according to size of perforation and PRP PRP: platelet-rich plasma

Perforation size	PRP not used	PRP used	Total
Small	4 (36.4%)	7 (63.6%)	11 (18.3%)
Moderate	13 (52.0%)	12 (48.0%)	25 (41.7%)
Large	9 (50.0%)	9 (50.0%)	18 (30.0%)
Subtotal	4 (66.7%)	2 (33.3%)	6 (10.0%)
Total	30 (50.0%)	30 (50.0%)	60 (100.0%)

Table 6 shows that PRP improves graft acceptance, especially for moderate perforations in postauricular tympanoplasty. Significant results (p = 0.012) suggest PRP positively impacts graft outcomes.

**Table 6:** Anatomical outcomes of tympanic membrane graft acceptance: perforation size, PRP usage, and surgical approach analysis PRP: platelet-rich plasma; p-value: significant value

Perforation size	PRP usage	Endoscopic tympanoplasty			Postauricular tympanoplasty			p-value
		Accepted	Not accepted	Partially accepted	Accepted	Not accepted	Partially accepted	
Small	Not used	2 (50.0%)	0 (0.0%)	0 (0.0%)	2 (50.0%)	0 (0.0%)	0 (0.0%)	0.012
	Used	2 (28.6%)	0 (0.0%)	0 (0.0%)	5 (71.4%)	0 (0.0%)	0 (0.0%)	
Moderate	Not used	5 (33.3%)	0 (0.0%)	0 (0.0%)	4 (26.7%)	0 (0.0%)	3 (20.0%)	
	Used	6 (36.4%)	0 (0.0%)	0 (0.0%)	4 (24.2%)	1 (6.1%)	0 (0.0%)	
Large	Not used	4 (26.7%)	1 (6.7%)	1 (6.7%)	2 (13.3%)	1 (6.7%)	1 (6.7%)	
	Used	4 (30.8%)	0 (0.0%)	0 (0.0%)	4 (30.8%)	0 (0.0%)	1 (7.7%)	
Subtotal	Not used	1 (50.0%)	0 (0.0%)	1 (50.0%)	2 (50.0%)	0 (0.0%)	0 (0.0%)	
	Used	3 (100.0%)	0 (0.0%)	0 (0.0%)	0 (0.0%)	0 (0.0%)	0 (0.0%)	
Total		27	1	2	23	2	5	

Table 7 shows that endoscopic tympanoplasty results in greater hearing gain than conventional tympanoplasty, with significant improvements for small and large perforations (p = 0.014).

**Table 7:** Functional outcome and surgical approaches p-value: significant value

Perforation size	Endoscopic			Conventional			p-value
	Preoperative	Postoperative	Hearing gain	Preoperative	Postoperative	Hearing gain	
Small	39.50 ± 5.97	34.0 ± 5.74	5.5	39.14 ± 10.86	34.85 ± 10.44	4.2	0.014
Moderate	54.0 ± 13.17	43.66 ± 12.94	10.4	50.12 ± 13.03	42.96 ± 13.29	7.2	
Large	55.09 ± 9.59	44.0 ± 8.76	11	53.36 ± 16.11	45.25 ± 17.21	8.1	
Subtotal	64.75 ± 21.71	50.03 ± 18.19	14.7	56.0 ± 7.07	43.0 ± 5.65	13	
Average hearing gain			10.4			8.1	

Table 8 shows PRP usage results in greater hearing gains post-surgery across all perforation sizes, with the most significant improvement observed in moderate hearing loss cases (p = 0.014).

**Table 8:** Functional outcome and PRP p-value: significant value

Perforation size	Non-PRP			PRP			p-value
	Preoperative	Postoperative	Hearing gain	Preoperative	Postoperative	Hearing gain	
Small	34.50 ± 6.40	31.25 ± 5.50	3.25	41.0 ± 10.62	37.66 ± 10.21	3.34	0.014
Moderate	44.27 ± 9.18	41.65 ± 9.33	2.62	57.91 ± 13.74	53.16 ± 14.11	4.75	
Large	47.38 ± 10.41	44.88 ± 10.12	2.5	52.81 ± 14.44	49.11 ± 15.36	3.7	
Subtotal	50.0 ± 9.93	44.75 ± 10.84	5.25	69.0 ± 22.06	61.66 ± 18.03	7.34	

## Discussion

This study aimed to evaluate the outcomes of tympanoplasty based on various factors, including age, hearing loss severity, surgical approach, and the use of PRP. The results provide insights into how these variables influence surgical success and functional recovery. The distribution of patients by age shows a higher prevalence of tympanoplasty in individuals aged 19-50 years, with endoscopic tympanoplasty being more common in younger patients and postauricular tympanoplasty in older individuals. This finding aligns with previous research indicating that younger patients often opt for more advanced surgical techniques. In contrast, older patients might prefer traditional methods due to various comorbidities and anatomical considerations [14,15].

A significant correlation was found between the degree of hearing loss and the size of the perforation (p = 0.001), which corroborates previous studies that have shown larger perforations are associated with more severe hearing loss [16,17]. This correlation is critical for tailoring surgical interventions and predicting patient outcomes. The preference for endoscopic tympanoplasty in cases of moderate hearing loss and postauricular tympanoplasty for mild hearing loss suggests a tailored approach to surgical technique based on the severity of hearing impairment. This practice is supported by evidence that different surgical approaches can yield varied functional outcomes depending on the extent of hearing loss [18,19].

The results indicate that PRP usage is associated with improved outcomes, particularly in moderate and severe cases of hearing loss. The use of PRP significantly enhances graft acceptance rates, especially in moderate perforations treated with postauricular tympanoplasty (p = 0.012). This finding is consistent with literature suggesting that PRP can accelerate tissue healing and improve surgical outcomes by promoting cellular regeneration and reducing inflammation [20,5]. Endoscopic tympanoplasty demonstrated greater hearing gain than conventional tympanoplasty, especially in small and large perforations cases. This aligns with studies highlighting the advantages of endoscopic techniques in achieving better functional results due to more precise and less invasive procedures [6,21]. Additionally, PRP usage led to greater hearing improvements across all perforation sizes, with the most substantial gains observed in moderate cases. This reinforces the notion that PRP contributes positively to post-surgical hearing recovery [5].

Limitation

This study's limitations include a small sample size of 60 patients, which may affect the generalizability of the results. Conducted at a single tertiary care hospital, the findings may not apply to other settings. The three-month follow-up period may not capture long-term outcomes, and PRP preparation and surgical experience variability could impact results. Additionally, subjective assessment methods may introduce variability. Larger, multicenter studies with extended follow-up are needed to validate these findings and address these limitations.

## Conclusions

This study underscores the superior efficacy of ET over conventional PA in managing COM. ET not only achieved higher rates of anatomical success but also resulted in more substantial improvements in hearing, particularly when combined with PRP. The enhanced graft acceptance and greater hearing gains observed with ET highlight its advantages as a minimally invasive approach, making it a preferred surgical option for tympanoplasty. Additionally, the application of PRP further contributed to improved outcomes, reinforcing its beneficial role in tympanoplasty procedures. These findings advocate for the increased adoption of ET and PRP in clinical practice, promising better overall patient outcomes and recovery in tympanoplasty surgeries.

## References

[1] Rosario DC, Mendez MD (2024). Chronic suppurative otitis. https://www.ncbi.nlm.nih.gov/books/NBK554592/.

[2] Brar S, Watters C, Winters R (2024). Tympanoplasty. https://www.ncbi.nlm.nih.gov/books/NBK565863/.

[3] Cho YS, Park MH, Han UG, Son SE, Moon IJ (2022). Outcomes and learning curve of endoscopic tympanoplasty: a retrospective analysis of 376 patients. Laryngoscope Investig Otolaryngol.

[4] Akyigit A, Sakallıoglu O, Karlidag T (2017). Endoscopic tympanoplasty. J Otol.

[5] Taneja MK (2020). Role of platelet rich plasma in tympanoplasty. Indian J Otolaryngol Head Neck Surg.

[6] Choi N, Noh Y, Park W (2017). Comparison of endoscopic tympanoplasty to microscopic tympanoplasty. Clin Exp Otorhinolaryngol.

[7] Beckmann S, Anschuetz L (2021). Minimally invasive tympanoplasty: review of outcomes and technical refinements. Oper Tech Otolayngol Head Neck Surg.

[8] Kawale M, Landge S, Garg D, Kanani K (2023). Endoscopic versus microscopic type 1 tympanoplasty (myringoplasty) in a rural tertiary care hospital in india: a retrospective comparative study. Cureus.

[9] Gunaratnam C, Bernstein M (2018). Factors affecting surgical decision-making—a qualitative study. Rambam Maimonides Med J.

[10] (2024). Tuning Fork Tests - Rinne’s test, Weber’s test, Schwabach test. https://www.entlecture.com/tuning-fork-tests/.

[11] (2024). ALPS AD2000 Hearing Aid Audiometers. https://www.hospitalstore.com/alps-ad2000-hearing-aid-audiometers/.

[12] (2024). ENT/Otorhinolaryngology | KARL STORZ Endoskope. https://www.karlstorz.com/de/en/ent-otorhinolaryngology.htm.

[13] (2024). IBM SPSS Statistics. https://www.ibm.com/products/spss-statistics.

[14] Agrawal A, Bhargava P (2017). Comparative evaluation of tympanoplasty with or without mastoidectomy in treatment of chronic suppurative otitis media tubotympanic type. Indian J Otolaryngol Head Neck Surg.

[15] Yang Q, Wang B, Zhang J, Liu H, Xu M, Zhang W (2022). Comparison of endoscopic and microscopic tympanoplasty in patients with chronic otitis media. Eur Arch Otorhinolaryngol.

[16] Bhutta MF, Thornton RB, Kirkham LS, Kerschner JE, Cheeseman MT (2017). Understanding the aetiology and resolution of chronic otitis media from animal and human studies. Dis Model Mech.

[17] Fitzgerald DC (2001). Nasopharyngeal abscess and facial paralysis as complications of petrous apicitis: a case report. Ear Nose Throat J.

[18] Bianchini AJ, Berlitz VG, Mocelin AG, Ribeiro JF, Keruk JG, Hamerschmidt R (2023). Endoscopic or microscopic tympanoplasty advantages and disadvantages: a theory domain systematic review. Int Arch Otorhinolaryngol.

[19] Walker EA, Holte L, McCreery RW, Spratford M, Page T, Moeller MP (2015). The influence of hearing aid use on outcomes of children with mild hearing loss. J Speech Lang Hear Res.

[20] Huang J, Shi Y, Wu L, Lv C, Hu Y, Shen Y (2021). Comparative efficacy of platelet-rich plasma applied in myringoplasty: a systematic review and meta-analysis. PLoS One.

[21] Gkrinia E, Ntziovara AM, Brotis AG, Tzimkas-Dakis K, Saratziotis A, Korais C, Hajiioannou J (2024). Endoscopic versus microscopic tympanoplasty: a systematic review and metanalysis. Laryngoscope.

